# Correlation between quality and geographical origins of Leonuri Herba revealed by the qualitative fingerprint profiling and quantitative determination of chemical components

**DOI:** 10.1186/s13020-022-00592-w

**Published:** 2022-04-12

**Authors:** Kelly Yinching Lam, Yinghao Wang, Tszking Lam, Chuenfai Ku, Wingping Yeung, Zhongzhen Zhao

**Affiliations:** 1 School of Chinese Medicine, Hong Kong Baptist University, Hong Kong, China; 2 Department of Chinese Materia Medica, College of Pharmacy, Fujian University of Traditional Chinese Medicine, Fuzhou, China

## Abstract

**Background:**

Leonuri Herba (Yimucao) is a very common Chinese herbs for treating menstrual and maternal diseases for thousands of years in China. However, the herb collected in different origins was easily found in the markets which induce the unstable quality for clinic use. In this study, a comprehensive strategy of using multiple chromatographic analysis and chemometric analysis was firstly investigated for chemical discrimination of Leonuri Herba from different geographical origins.

**Methods:**

UHPLC-QTOF-MS/MS was applied to identify the peaks of Leonuri Herba and chemical fingerprints were established in 30 batches from different geographical origins. Meanwhile, dissimilarities of chemical compositions among different origins were further investigated by principal component analysis and cluster analysis. And a quantitative UHPLC-QTOF-MS/MS approach were established to investigate the potential marker for quality control of Leonuri Herba.

**Results:**

A total of 49 chromatographic peaks of Leonuri Herba were identified by UHPLC-QTOF-MS/MS. Leonuri Herba were classified into four categories, and eight major compounds detected could be used as chemical markers for discrimination. Also, the eight components, including leonurine, 4',5-dihydroxy-7-methoxyflavone, rutin, hyperoside, apigenin, quercetin, kaempferol and salicylic acid, were simultaneously quantified using the extracting ion mode of UHPLC-QTOF-MS/MS.

**Conclusion:**

The current strategy not only clearly expounded the correlation between quality and geographical origins of Leonuri Herba, but also provided a fast, accurate and comprehensive qualitative and quantitative method for assessing the quality of Leonuri Herba.

**Supplementary Information:**

The online version contains supplementary material available at 10.1186/s13020-022-00592-w.

## Background

Leonuri Herba (Yimucao) is the aerial part of *Leonurus japonicus* Houtt., which is a very common Chinese herb for treating menstrual and maternal diseases. It has been used for thousands of years in Chinese Medicine, and it is recognized to be a non-toxic herb [1]. *Leonurus japonicus* Houtt. grows on wastelands of mountains and plains, rand, grasslands and streamside. It was distributed or cultivated in all parts of China. The medicinal materials are mainly produced in Henan (Songxian and Luanzhou), Anhui (Liuan and Bengbu), Sichuan (Wenjiang), Jiangsu (Nanjing), Zhejiang (Fenghua) [2]. Besides, it is also native to other countries such as Europe, North America, Japan and Malaysia [3]. According to Chinese Pharmacopoeia [4], the dried herb is collected in summer before flowering and dried under the sun to obtain Herba Leonuri. However, the herb collected in different stages with the varied proportion of leaves and stems was easily found in the markets which induce the unstable quality for clinic use. Thus, the quality of this traditional Chinese Materia Medica has already attracted public concern. Leonuri Herba has plenty of pharmacological effects for treating human diseases [5], especially in gynecopathy [6], as well as other functions with its low toxic property [7]. Thus, it has been using for a long time as traditional Chinese medicine (TCM). A substantial number of studies have been conducted to investigate its active components and related mechanisms [5].

Leonuri Herba is not a geo-authentic herb (daodi yaochai). It is difficult to determine the quality of Leonuri Herba in different geographical origins. Furthermore, different harvesting period of Leonuri Herba is also one of the major factors affecting the quality. Nowadays, Leonuri Herba at seedling stage is commonly used in Hong Kong market while the mature one is widely used in mainland China. These situations show the necessity and importance to clarify which growing stage and geographical origins that should be chosen. There are around 140 compounds discovered in Leonuri Herba, alkaloids, diterpenes and flavones are its main chemicals [1]. Apart from leonurine and stachydrine, the studies of other components such as diterpenes and flavones that monitoring the quality of Leonuri Herba is still deficient [3]. This leads to the significance of analyzing the quality of Leonuri Herba by other chemical components.

In this study, we investigated whether and how the quality of Leonuri Herba correlates with its geographical origins by combining UHPLC-QTOF-MS/MS-based qualitative fingerprint profiling and quantitative determination of potential markers. The data obtained were processed by multivariate statistical analysis, including hierarchical cluster analysis (HCA), principal component analysis (PCA) and supervised orthogonal partial least squared discriminant analysis (OPLS-DA), to evaluate the differences in quality of these samples. Finally, in order to better understand the differences from quantitative levels, potential markers that played key roles in differentiating Leonuri Herba from different locations, were simultaneously determined in different samples.

## Materials and methods

### Herbal materials

Thirty batches of Leonuri Herba were acquired for this study. Of these, ten batches were obtained from the Hong Kong TCM market, other batches were purchased directly from certified production regions in China, as specified in Table 1. Thirty batches of Leonuri Herba were all authenticated by Prof. Zhongzhen Zhao from the School of Chinese Medicine (SCM), Hong Kong Baptist University (HKBU). Samples were dried, ground, then sifted through a 24-mesh sieve. Voucher specimens were deposited in SCM of HKBU (Additional file 1).

**Table 1:** The batch number and geographic habitats of 30 samples of Leonuri Herba

Batch no	Sample code	Name	Growing stage	Habitats	Stem/%	Leaf/%
S1	HB1	Leonuri Herba	Mature	Hubei, China	67.90	32.10
S2	HB2	Leonuri Herba	Seedling	Hubei, China	20.25	79.75
S3	HB3	Leonuri Herba	Mature	Hubei, China	69.48	30.52
S4	GD1	Leonuri Herba	Seedling	Guangdong, China	30.05	69.95
S5	HB4	Leonuri Herba	Flowering	Hubei, China	23.08	76.92
S6	GD2	Leonuri Herba	Seedling	Guangdong, China	18.44	81.56
S7	GD3	Leonuri Herba	Seedling	Guangdong, China	29.68	70.32
S8	HB5	Leonuri Herba	Seedling	Hubei, China	34.24	65.76
S9	HB6	Leonuri Herba	Flowering	Hubei, China	31.06	68.94
S10	HB7	Leonuri Herba	Seedling	Hubei, China	25.54	74.46
S11	HN1	Leonuri Herba	Flowering	Henan, China	62.26	37.74
S12	HN2	Leonuri Herba	Flowering	Henan, China	61.68	38.32
S13	HN3	Leonuri Herba	Flowering	Henan, China	52.12	47.88
S14	HN4	Leonuri Herba	Flowering	Henan, China	66.87	33.13
S15	HN5	Leonuri Herba	Flowering	Henan, China	63.37	36.63
S16	AH1	Leonuri Herba	Flowering	Anhui, China	63.29	36.71
S17	AH2	Leonuri Herba	Flowering	Anhui, China	53.69	46.31
S18	AH3	Leonuri Herba	Flowering	Anhui, China	66.13	33.87
S19	AH4	Leonuri Herba	Flowering	Anhui, China	38.12	61.88
S20	YN1	Leonuri Herba	Flowering	Yunnan, China	58.84	41.16
S21	YN2	Leonuri Herba	Flowering	Yunnan, China	47.32	52.68
S22	YN3	Leonuri Herba	Flowering	Yunnan, China	53.26	46.74
S23	YN4	Leonuri Herba	Flowering	Yunnan, China	56.73	43.27
S24	ZJ1	Leonuri Herba	Flowering	Zhejiang, China	40.64	59.36
S25	ZJ2	Leonuri Herba	Flowering	Zhejiang, China	56.63	43.37
S26	SC1	Leonuri Herba	Flowering	Sichuan, China	48.23	51.77
S27	SC2	Leonuri Herba	Flowering	Sichuan, China	52.92	47.08
S28	SC3	Leonuri Herba	Flowering	Sichuan, China	72.61	27.39
S29	SC4	Leonuri Herba	Flowering	Sichuan, China	57.52	42.48
S30	ZJ3	Leonuri Herba	Flowering	Zhejiang, China	40.45	59.55

### Instrument, chemicals and reagents

Chemical markers of leonurine, rutin, 4',5-dihydroxy-7-methoxyflavone, hyperoside, salicylic acid, kaempferol were obtained from Shanghai Tauto Biotech Co. Ltd. (Shanghai, China). Chemical marker of apigenin was purchased from Chengdu Mansite Pharmacetical Co. Ltd. (Sichuan, China). Chemical marker of quercetin was obtained from the National Institute of Control of Pharmaceutical and Biological Products (Beijing, China). The purity of each chemical marker was above 95%. Methanol and acetonitrile for UHPLC-QTOF-MS/MS analysis were obtained from Merck (Darmstadt, Germany). Ultrapure water was prepared by a Milli-Q water purification system (Millipore, Bedford, MA, USA).

The analyses were performed on UHPLC (Agilent Technologies Inc., Palo Alto, CA, USA), Agilent 6540 ultra-high-definition accurate mass quadrupole time-of-flight spectrometer (Agilent Technologies Inc., Wilmington, DE, USA).

### Sample extraction

Powdered sample (0.5 g) accurately weighed, was extracted with 5 mL of methanol for 60 min at 60 °C in an ultrasonic water bath (300 W), and then cooled to room temperature. After compensating the lost weight of methanol, the extracted solution was filtered through a 0.22 μm PTFE syringe filter UHPLC fingerprint and identification analysis by UHPLC-PDA-QTOF-MS/MS [8].

### UHPLC-QTOF-MS/MS identification

According to the research reports on the chemical components of Leonuri Herba, 121 chemical compounds are collected from Leonuri Herba. Agilent’s “Mass Hunter PCDL Manager” software is used to calculate the relatively molecular mass accurately and establish a database of known chemical components of Leonuri Herba.

### Chromatographic conditions

Waters ACQUITY UPLC® BEH-C18 analytical column (2.1 mm × 100 mm, I.D. 1.7 µm); VanGuardTM BEH-C18 guard column (2.1 mm × 5 mm, I.D. 1.7 µm); Mobile phase: 0.1% FA (A) and acetonitrile (B); Linear gradient elution program: 0–8 min, 25% B; 8–16 min, 25–75% B; 16–18 min, 75–100% B; 18–21 min, 100% B; 21 min, 2% B; 21–24 min, 2% B; Flow rate: 0.35 mL/min; Column temperature: 40 ℃; Injection volume: 2 µL.

### Mass spectrometry conditions

Mode: positive and negative(scanning from 100 to 900 m/z); Dry gas temperature 300 ℃; Dry nitrogen gas flow rate 8 L/min; Nebulizer pressure 40 psi; Vcap 3500; Nozzle voltage 500 V; Fragmentor voltage 120 V. The mass spectrum was calibrated by Tune mix every time (positive mode, 118–1521 m/z; negative mode, 112–1634 m/z).

### Fingerprint similarity evaluation

According to the UHPLC-MS/MS spectra of 30 samples, the common chromatographic fingerprint peaks and internal reference peaks contained in each batch of samples were confirmed. The Similarity evaluation system for chromatographic fingerprint of TCM was used to establish the fingerprint of 30 batches of Leonuri Herba. The similarity was calculated by the angle cosine method.

### Hierarchical clustering analysis and principal component analysis

For HCA, the common fingerprint peak area of Leonuri Herba from different origins and batches are used as the source data of hierarchical clustering analysis. The hierarchical clustering algorithm in microarray analysis software (MeV 4.7.4) is used. For PCA, the common fingerprint peak area of Leonuri Herba fingerprints from different origins and batches are used as the source data of PCA by using factor analysis in SPSS 20.0(SPSS Corporation, Armonk, NY, USA).

### Quantitative analysis

The accurately weighed powdered samples of 30 batches (0.5 g) were respectively extracted with methanol (2.5 mL) for 60 min, and then cooled to 25 ℃. After centrifugation at 3500*g* for 10 min, the supernatant was transferred to a 10 mL volumetric flask. This process was repeated twice and then the volume was adjusted to the calibration mark with methanol. The extracted solution was filtered through a 0.22 μm PTFE syringe filter before analysis. The standards were accurately weighed and dissolved into methanol. A series of standard solutions for constructing working standard curves was prepared by diluting the mixed stock standard solution with methanol, and a calibration curve was established by plotting peak areas (y axis) versus concentrations (x axis).

### Statistical analysis

Peaks above the S/N ratio of the chromatogram were labeled and manually integrated using version 7.2 of the Chromeleon Chromatography Data System software (Thermo Fisher Scientific) to distinguish between the different batches. PCA and OPLS-DA were performed using the software SIMCA-P Version 13.0 (Umetrics). Summary data were expressed as the mean ± standard deviation (SD) for *n* = 3–7.

## Results and discussion

### Component authentication

**Fig. 1:**
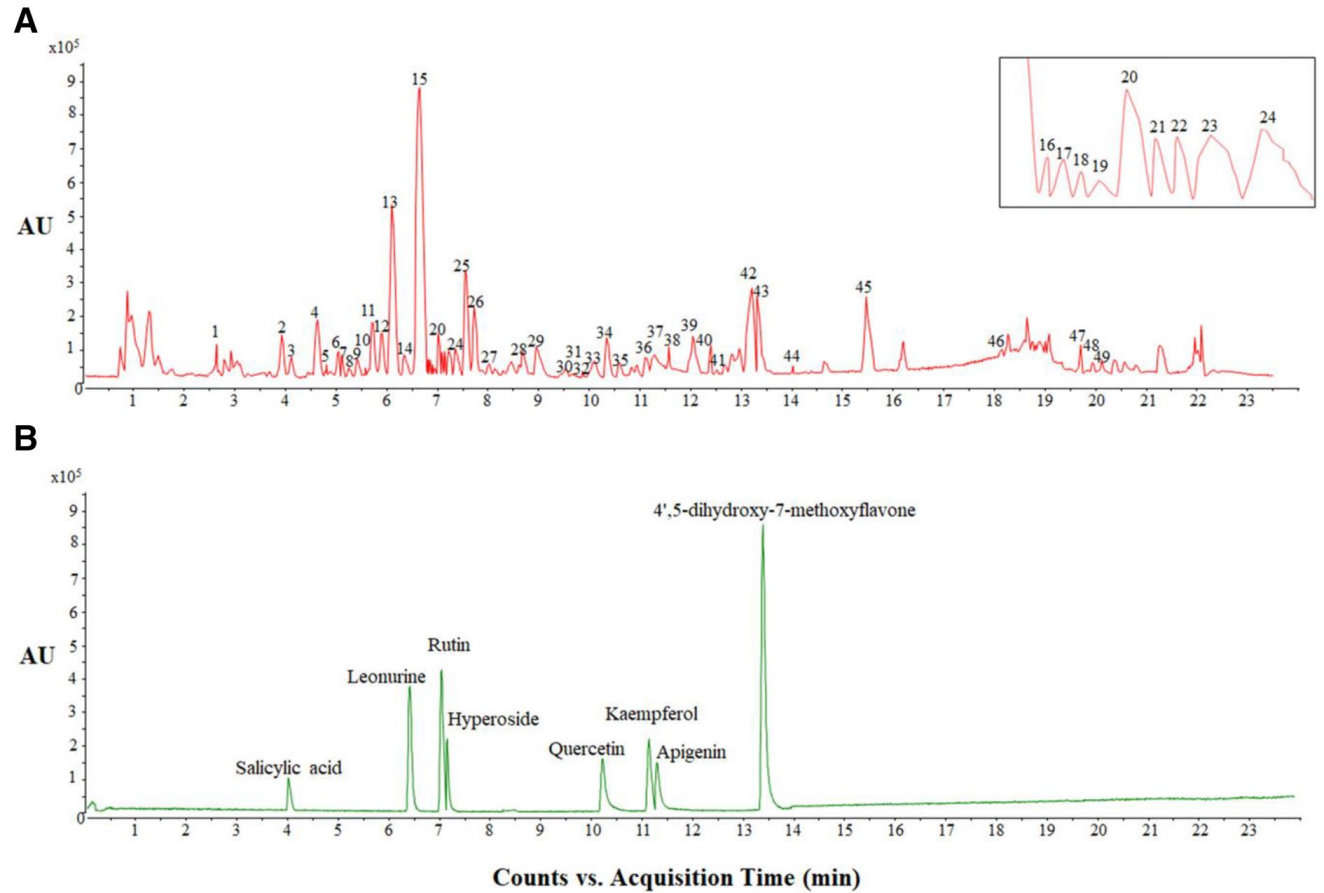
The BPC of Leonuri Herba (**A**) and 8 chemical marker (**B**) in negative mode

Figure 1A shows the Base peak chromatogram (BPC) of Leonuri Herba in negative mode. According to TR, Mass, (−)-ESI–MS/MS Fragment Ions and compared with the reference and standards, 49 components were identified or preliminarily identified. There were 2 alkaloids, 18 flavonoids, 7 Terpenoids, 8 aromatic acids and 14 other classes (Table 2).

**Table 2:** Structural information of compounds characterized in the Leonuri Herba by negative-UHPLC-QTOF-MS/MS

	t_R_ (min)	MF	Product ion	Mass	Mass (Tgt)	Diff (ppm)	(−)-ESI–MS/MS Fragment Ions (*m/z*)	Identification [Reference]
1	2.630	C_9_H_11_NO_2_	[M-H]^−^	165.0797	165.0790	4.50	57.0355	Phenylalanine [6]
2	3.838	C_11_H_12_N_2_O_2_	[M+COOH]^−^	204.0908	204.0899	4.43		Tryptophan [6]
3	4.14	C_7_H_6_O_3_	[M-H]^−^	138.0324	138.0317	5.32		Salicylic acid [9]
4	4.548	C_9_H_10_O_3_	[M+COOH]^−^	166.0640	166.0630	5.97	150.0330, 72.9938	Ethyl 4-hydroxybenzoate [10]
5	4.764	C_14_H_20_O_9_	[M-H]^−^	332.1118	332.1107	3.25	316.0867, 197.0473, 61.9892	2,6-Dimethoxy-4-hydroxyphenol-1-*O*-β-d-glucopyranoside [11]
6	4.989	C_27_H_42_O_17_	[M-H]^−^	638.2439	638.2422	2.60	578.2197, 461.2364, 255.2342, 89.025	Leonoside F [12]
7	5.223	C_7_H_6_O_2_	[M-H]^−^	122.0376	122.0368	6.49	167.0335, 108.0221	Benzoic acid [13]
8	5.340	C_8_H_8_O_3_	[M+COOH]^−^	152.0483	152.0473	6.41	123.0096	Vanillin [14]
9	5.341	C_9_H_10_O_5_	[M-H]^−^	198.0538	198.0528	5.02	123.0096	Syringic acid [13]
10	5.660	C_11_H_14_O_5_	[M+COOH]^−^	226.0859	226.0841	7.82	256.0593, 123.0099, 59.0140	3-Hydroxyl-1-(4-hydroxy-3,5-dimethoxyphenyl)-1-propanone [1]
11	5.865	C_19_H_32_O_8_	[M+COOH]^−^	388.2109	388.2097	3.10	401.1477, 197.0454, 61.9893	Staphylionoside E [12]
12	6.533	C_8_H_8_O_4_	[M-H]^−^	168.0430	168.0423	4.45	108.0221	Vanillic acid [14]
13	6.542	C_7_H_6_O_2_	[M-H]^−^	122.0376	122.0368	6.51	167.0335, 108.0221	4-Hydroxybenzaldehyde [11]
14	6.563	C_19_H_30_O_8_	[M-H]^−^	386.1948	386.1941	1.91	189.1284, 85.0309	Citroside A [12]
15	6.617	C_14_H_21_N_3_O_5_	[M-H]^−^	311.1497	311.1481	4.96	280.0956, 95.0148	Leonurine [15]
16	6.646	C_27_H_30_O_16_	[M-H]^−^	610.1556	610.1534	3.69	447.0039, 300.0293, 151.0054	Quercetin-3-*O*-robinoside [16]
17	6.746	C_13_H_20_O_3_	[M+COOH]^−^	224.1426	224.1412	5.96		(2S,5S)-2-Hydroxy-2,6,10,10-tetramethyl-1-oxaspiro-[4.5]dex-6-en-8-one [1]
18	6.884	C_21_H_20_O_11_	[M+COOH]^−^	448.1027	448.1006	4.73	284.0341, 61.9894	Kaempferol-3-*O*-β-d-glucopyranoside [16]
19	6.968	C_27_H_30_O_16_	[M-H]^−^	610.1558	610.1534	3.94	447.1139, 300.0294, 151.0055	Rutin [13]
20	6.997	C_10_H_10_O_4_	[M-H]^−^	194.0591	194.0579	5.97	134.0387, 58.0307	Trans-ferulic acid [11]
21	7.173	C_21_H_20_O_12_	[M-H]^−^	464.0978	464.0955	4.93	300.0293, 61.9891	Hyperoside [16]
22	7.306	C_21_H_20_O_12_	[M-H]^−^	464.0976	464.0955	4.67	300.0293, 151.0048, 61.9891	Quercetin-3-*O*-β-d-glucopyranoside [16]
23	7.335	C_27_H_30_O_15_	[M-H]^−^	594.1607	594.1585	3.76	463.0907, 284.0344, 61.9891	Kaempferol-3-neohesperidoside [16]
24	7.494	C_34_H_44_O_19_	[M-H]^−^	756.2495	756.2477	2.37	593.2095, 461.1679, 357.1262, 223.0608, 161.0254	Lavandulifolioside [12]
25	7.507	C_27_H_30_O_15_	[M-H]^−^	594.1607	594.1585	3.79	387.1094, 284.0344, 61.9891	Kaempferol-3-*O*-β-robinobinoside [13]
26	7.832	C_27_H_30_O_15_	[M-H]^−^	594.1606	594.1585	3.56	387.1094, 284.0344, 61.9891	Kaempferol-3-*O*-rutinoside [11]
27	8.083	C_21_H_20_O_11_	[M-H]^−^	448.1026	448.1006	4.66	284.0341, 61.9894	Kaempferol-3-*O*-β-d-galactopyranoside [16]
28	8.493	C_15_H_20_O_3_	[M-H]^−^	248.1427	248.1412	6.00		Arteannuin B [17]
29	8.903	C_36_H_38_O_20_	[M-H]^−^	790.1983	790.1956	3.42	609.1483, 473.1289, 379.1785, 127.0408	2'''-syringylrutin [18]
30	9.635	C_12_H_20_O_5_	[M-H]^−^	244.1327	244.1311	6.47	207.1044, 71.0506	(E)-4-Hydroxy-dodec-2-enedioic acid [1]
31	9.693	C_21_H_32_O_8_	[M+COOH]^−^	412.2116	412.2097	4.67	327.1466, 61.9893	7α(H)-Eudesmane-4,11(12)-diene-3-one-2β-hydroxy-13-β-D-glucopyranoside [12]
32	10.16	C1_7_H_30_O_3_	[M+COOH]^−^	282.2207	282.2195	4.37		(-)-(1S*,2S*,3R*)-3-Ethoxycupar-5-ene-1,2-diol [1]
33	10.191	C_15_H_10_O_7_	[M-H]^−^	302.0441	302.0427	4.93	151.0046	quercetin [19]
34	10.308	C_30_H_26_O_13_	[M-H]^−^	594.1395	594.1373	3.66	447.0934, 285.0418, 145.0305	tiliroside [16]
35	10.542	C_30_H_26_O_13_	[M-H]^−^	594.1396	594.1373	3.84	447.0630, 285.0417, 145.0304	Kaempferol-3-*O*-( 6''-*O*-cis-p-coumaroyl) -β-d-glucopyranoside [11, 18]
36	11.216	C_15_H_10_O_6_	[M-H]^−^	286.0493	286.0477	5.49	65.0044	Kaempferol [20]
37	11.333	C_10_H_18_O_2_	[M+COOH]^−^	170.1319	170.1307	6.98	153.1285, 68.9956	2,6-dimethyl-2E,7-octadiene-1,6-diol [16]
38	11.45	C_15_H_10_O_5_	[M+COOCH_3_]^−^	270.0545	270.0528	6.24		Apigenin [11]
39	12.036	C_13_H_16_O	[M+COOCH_3_]^−^	188.1192	188.1201	-5.09	185.9999, 75.0097	2-(1-Oxopentyl)-benzoic acid methyl ester [16]
40	12.241	C_15_H_30_O_2_	[M+COOH]^−^	242.2261	242.2246	6.09		Methyl tetradecanoate [16]
41	12.563	C_16_H_12_O_5_	[M-H]^−^	284.0698	284.0685	4.58	268.0395, 117.0356	Wogonin [20]
42	13.244	C_16_H_12_O_5_	[M-H]^−^	284.0700	284.0685	5.51	268.0395, 117.0356	4',5-Dihydroxy-7-methoxyflavone [18]
43	13.444	C_15_H_10_O_4_	[M+COOH]^−^	254.0593	254.0579	5.61	284.0342, 133.0305	Daidzein [18]
44	13.978	C_21_H_32_O_6_	[M+COOH]^−^	380.2201	380.2199	0.50	263.1668, 61.9892	(-)-(5S,7R,8R,9R,10S,13S,15R)-7-hydroxy-15-methoxy-9,13;15,16-diepoxylabdan-6,16-dione [1]
45	15.502	C_15_H_24_O_4_	[M-H]^−^	268.1687	268.1675	4.72		7α(H),10α-eudesm-4-en-3one-2β,11,12-triol [14]
46	18.29	C_20_H_28_O_4_	[M+COOCH_3_]^−^	332.1981	332.1988	-2.01		(-)-3α-Acetoxy-6β-hydroxy-15,16-dinorlabd-8(9)-ene-13-yne-7-one [18]
47	19.769	C_30_H_48_O_4_	[M-H]^−^	472.3571	472.3553	3.80	409.3488, 171.1035, 61.9893	Messagenic acid [21]
48	19.881	C_29_H_46_O_2_	[M+COOH]^−^	426.3516	426.3498	4.25	409.3488, 171.1035, 61.9893	(24S)-Stigmast-4,28-diene-24-ol-3-one [21]
49	20.276	C_20_H_40_O	[M+COOCH_3_]^−^	296.3097	296.3079	6.14		Phytol [21]

### Fingerprint analysis

**Fig. 2:**
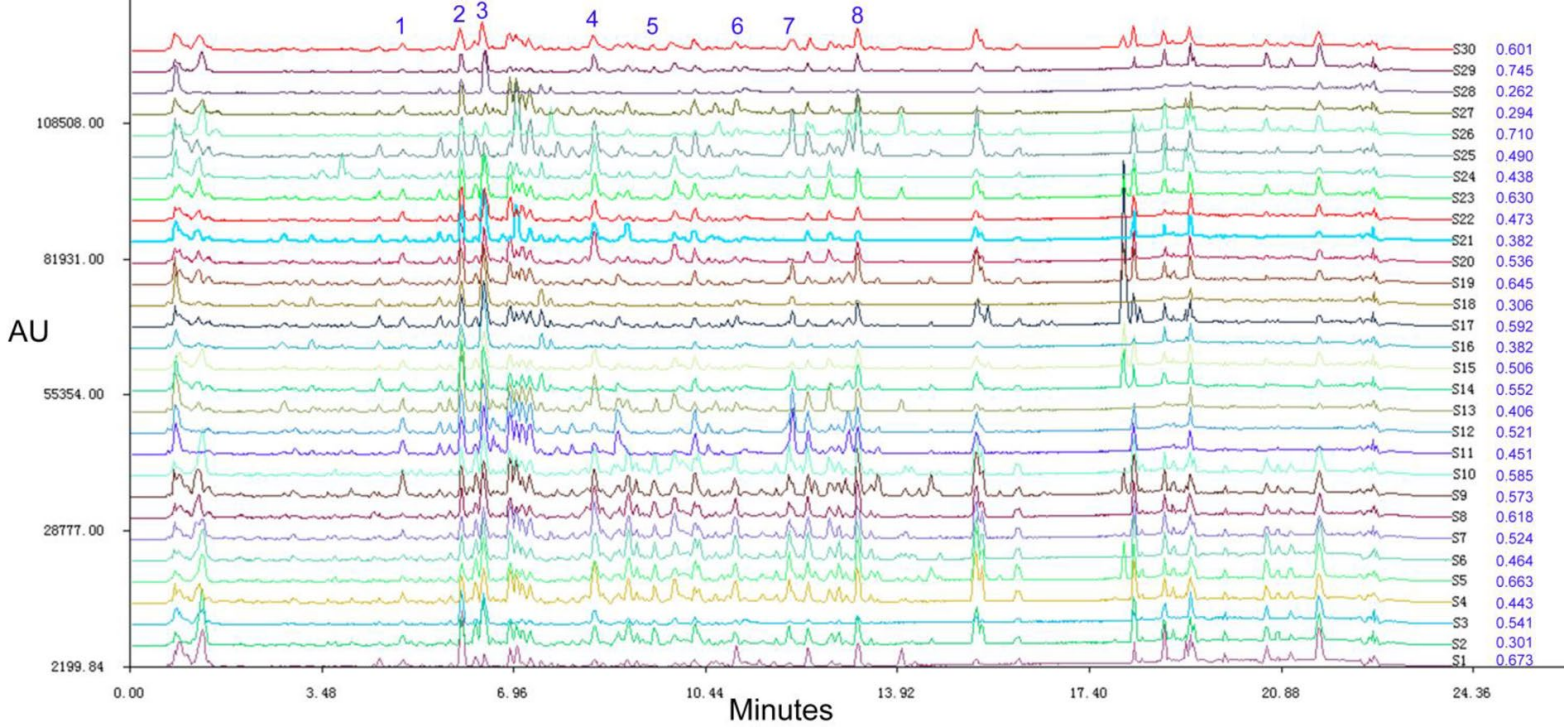
Overlay chromatograms of the 30 Leonuri Herba samples and the similarity of each chromatogram to their simulated mean chromatogram in blue. 1: salicylic acid; 2: leonurine; 3: rutin; 4: hyperoside; 5: quercetin; 6: Kaempferol; 7: apigenin; 8: 4',5-dihydroxy-7-methoxyflavone

According to the UHPLC-MS/MS spectrum of 30 batches of samples, the chromatographic peaks was showed within 24 min. Thirty-three of them were the common peaks to each batch of samples (Table 3). The total peak area of each batch of samples accounted for more than 85% of the total peak area and the reproducibility was good, which fulfilled the requirements of fingerprints. Thus, they were determined as common fingerprint peaks. The common fingerprint peak was determined by the standard retention time. When the retention time was 6.53 min, it was leonurine (Fig. 1B). Since the peak area of leonurine was relatively big, the peak time was moderate and the shape was good, it was selected as the reference peak. Using Similarity Evaluation System for Chromatographic Fingerprint of Traditional Chinese Medicine (2004A edition). Thirty batches of Leonuri Herba from different origins were introduced (Fig. 2) and the time width was 0.1 min, gaining the control fingerprint by median generation method. The similarity of 30 batches of samples was calculated by the angle cosine method. The result showed the similarity between each fingerprint and the control fingerprint was less than 0.90, indicating there was a big difference in each origin including Hubei, Guangdong, Henan, Anhui, Yunnan, Zhejiang and Sichuan. Thus, principal component and hierarchical cluster analysis were further analysed to clarify the relationship between origins and quality.

**Table 3:** Common fingerprint peaks of the fingerprint of Leonuri Herba in different origins

Peak No	Chemical type	Compounds	MF
C1	Alkaloid	Leonurine	C_14_H_21_N_3_O_5_
C2	Alkaloid	Tryptophan	C_11_H_12_N_2_O_2_
C3	Flavonoid	4',5-Dihydroxy-7-methoxyflavone	C_16_H_12_O_5_
C4	Flavonoid	Rutin	C_27_H_30_O_16_
C5	Flavonoid	Quercetin-3-*O*-robinoside	C_27_H_30_O_16_
C6	Flavonoid	Tiliroside	C_30_H_26_O_13_
C7	Flavonoid	Kaempferol-3-*O*-β-d-glucopyranoside	C_21_H_20_O_11_
C8	Flavonoid	Kaempferol-3-*O*-β-d-galactopyranoside	C_21_H_20_O_11_
C9	Flavonoid	Kaempferol-3-neohesperidoside	C_27_H_30_O_15_
C10	Flavonoid	Kaempferol-3-*O*-rutinoside	C_27_H_30_O_15_
C11	Flavonoid	Hyperoside	C_21_H_20_O_12_
C12	Flavonoid	Quercetin-3-*O*-β-d-glucopyranoside	C_21_H_20_O_12_
C13	Flavonoid	Daidzein	C_15_H_10_O_4_
C14	Flavonoid	2'''-Syringylrutin	C_36_H_38_O_20_
C15	Flavonoid	Apigenin	C_15_H_10_O_5_
C16	Flavonoid	Quercetin	C_15_H_10_O_7_
C17	Flavonoid	Kaempferol	C_15_H_10_O_6_
C18	Terpenoids	Staphylionoside E	C_19_H_32_O_8_
C19	Terpenoids	Citroside A	C_19_H_30_O_8_
C20	Terpenoids	7α(H)-Eudesmane-4,11(12)-diene-3-one-2β-hydroxy-13-β-d-glucopyranoside	C_21_H_32_O_8_
C21	Terpenoids	(-)-(5S,7R,8R,9R,10S,13S,15R)-7-hydroxy-15-methoxy-9,13;15,16-diepoxylabdan-6,16-dione	C_21_H_32_O_6_
C22	Aromatic acids	(E)-4-Hydroxy-dodec-2-enedioic acid	C_12_H_20_O_5_
C23	Other types	Phenylalanine	C_9_H_11_NO_2_
C24	Phenylethanoid glycosides	Lavandulifolioside	C_34_H_44_O_19_
C25	Other types	(−)-(1S*,2S*,3R*)-3-ethoxycupar-5-ene-1,2-diol	C_17_H_30_O_3_
C26	Other types	Messagenic acid	C_30_H_48_O_4_
C27	Other types	Phytol	C_20_H_40_O
C28	Aromatic acids	Syringic acid	C_9_H_10_O_5_
C29	Other types	2,6-Dimethoxy-4-hydroxyphenol-1-*O*-β-d-glucopyranoside	C_14_H_20_O_9_
C30	Aromatic acids	Salicylic acid	C_7_H_6_O_3_
C31	Other types	Ethyl 4-hydroxybenzoate	C_9_H_10_O_3_
C32	Flavonoid	Wogonin	C_16_H_12_O_5_
C33	Terpenoids	(−)-3α-Acetoxy-6β-hydroxy-15,16-dinorlabd-8(9)-ene-13-yne-7-one	C_20_H_28_O_4_

### Results of hierarchical clustering analysis (HCA)

**Fig. 3:**
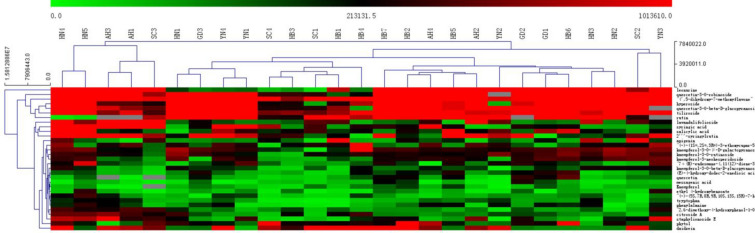
Hierarchical clustering analysis of Leonuri Herba from different origins and batches

The analysis showed the samples could be roughly clustered into two big groups. Five batches of Leonuri Herba from Henan, Anhui and Sichuan could be classified as one group, other samples could be classified as another group by the first chemical component clustering analysis. Based on the second to fifth chemical component clustering analysis, they were further clustered into small group: Anhui as a group; Yunnan as a group; Guangdong and Hubei as a group; Henan as a group. However, many factors such as harvest time, proportion of medical parts, soil, environment, and water were different, resulting in significant differences in chemical components of Leonuri Herba. Thus, it is necessary to find quality markers in different origins to clarify the relationship between habitat and quality. From the chemical composition clustering analysis, as shown in Fig. 3, the first component was leonurine. The second and third classes were quercetin-3-*O*-robinosideand 4',5-dihydroxy-7-methoxyflavone. The fourth class was hyperoside, quercetin-3-*O*-β-d-glucopyranoside and tiliroside. The fifth class was rutin, lavandulifolioside, syringic acid, salicylic acid and 2'''-syringylrutin. The sixth to eighth classes were apigenin. Other classes also included kaempferol, quercetin, tryptophan, 7α(H)-eudesmane-4,11(12)-diene-3-one-2β-hydroxy-13-β-d-glucopyranoside, etc. These different classes of components could be used to distinguish the quality of Leonuri Herba and as a basis for quantitative analysis.

Leonurine (C1), 4',5-dihydroxy-7-methoxyflavone (C3), rutin (C4), hyperoside (C11), apigenin (C15), quercetin (C16), kaempferol (C17) and salicylic acid (C30) were selected for further quantitative analysis of the indicator components.

### Results of multivariate statistical analysis

**Fig. 4:**
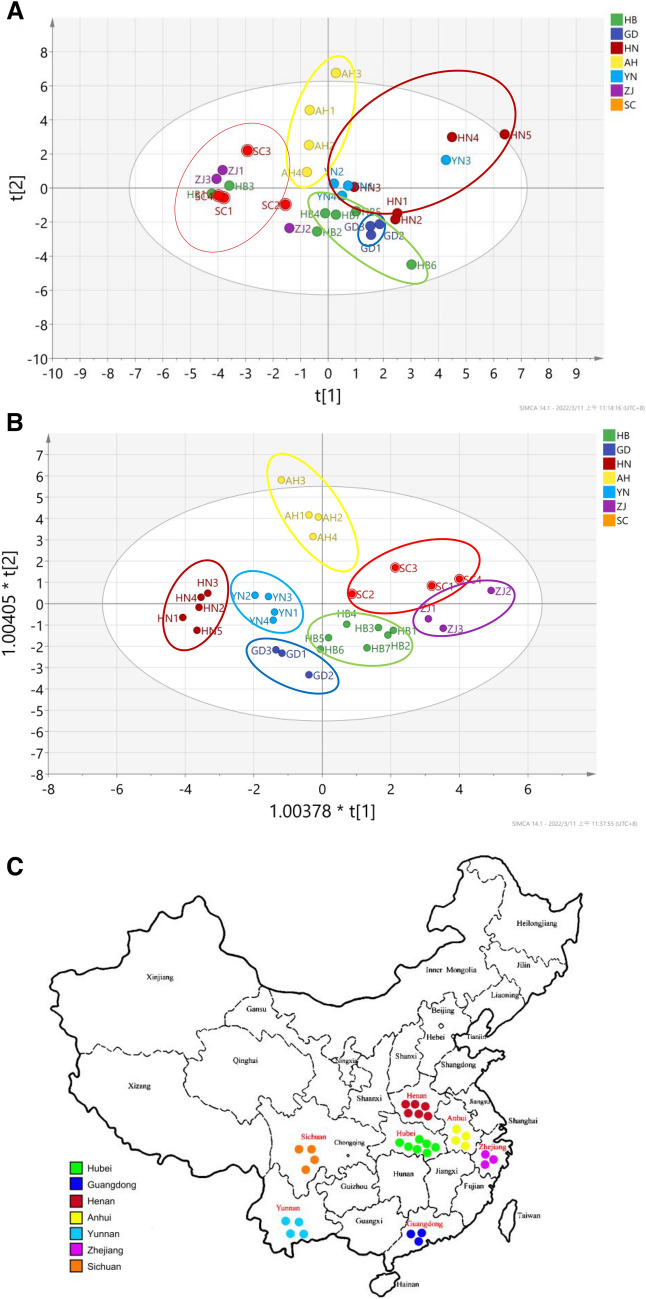
PCA/score plot (**A**) and OPLS-DA/score plot (**B**) based on the chemical profiling of 30 samples from different origins and the regional distribution of the corresponding 30 samples (**C**)

The common fingerprint peak area of Leonuri Herba fingerprints from different origins and batches were used as the source data of PCA by using factor analysis in SPSS 20.0. The results of SPSS analysis indicated that the first principal component should integrate the information of C1, C2, C3, C18, C21, C28, C29, C30 compounds. Thus, the chemical composition differences of different origins and batches of Leonuri Herba were mainly reflected to these chemical components. The results showed that the content of C1 (leonurine), C3 (4',5-dihydroxy-7-methoxyflavone) and C30 (salicylic acid) in Henan samples is higher, which was consistent with the cluster analysis results. The matrix coefficient of main components 1–8 in Leonuri Herba are shown in Fig. 4. In Fig. 4A, PCA divided Leonuri Herba from different origins into four parts: Henan, Yunnan as a large category; Zhejiang, Sichuan as a large category; Anhui as a large category; Guangdong, Hubei (at seedling and flowering stage) as a large category; Hubei (at mature stage) as a small category, which is different from the one at seedling and flowering stage. In order to further characterize the differences in chemical profiles among different Leonuri Herba samples, OPLS-DA, a supervised latent structures-discriminant analysis technique, which utilizes class information to maximize the separation between classes and minimize the discrimination between intra-groups, was performed to achieve better separation among different samples. The score plot of OPLS-DA indicated that all 30 samples were unambiguously classified into seven groups (Fig. 4B). Interestingly, these seven groups were highly consistent with the collection locations of these samples (Fig. 4C), which demonstrated that the chemical components of different samples are heavily influenced by growing area.

### Comprehensive evaluation analysis

The relationship formula between the principal component load matrix U, the factor load matrix A and the eigenvalue λ was U_i_ = A/ SQRT (λ_i_). By calculating the variables, eight eigenvectors U1-8 were obtained, and the expressions of the 8 principal components could be obtained, as follows.

$$\begin{aligned} {\text{Y}}_{{1}} =\, & 0.{\text{226X}}_{{1}} + 0.{\text{239X}}_{{2}} + 0.{\text{229X}}_{{3}} - 0.0{\text{45X}}_{{4}} + 0.{\text{213X}}_{{5}} + 0.{\text{161X}}_{{6}} + 0.{\text{215X}}_{{7}} + 0.{\text{145X}}_{{8}} + 0.{\text{189X}}_{{9}} + 0.{\text{147X}}_{{{1}0}} + 0.{\text{219X}}_{{{11}}} \\ &.{\text{182X}}_{{{12}}} - 0.0{\text{55X}}_{{{13}}} + 0.{\text{223X}}_{{{14}}} - 0.0{\text{53X}}_{{{15}}} + 0.0{\text{58X}}_{{{16}}} + 0.0{4}0{\text{X}}_{{{17}}} + 0.{\text{252X}}_{{{18}}} + 0.{\text{192X}}_{{{19}}} \\& + 0.{\text{183X}}_{{{2}0}} + 0.{\text{246X}}_{{{21}}} + 0.0{\text{87X}}_{{{22}}} + 0.{\text{213X}}_{{{23}}} + 0.{\text{152X}}_{{{24}}} + 0.0{\text{22X}}_{{{25}}} + 0.00{\text{4X}}_{{{26}}} - 0.0{\text{61X}}_{{{27}}} + 0.{\text{243X}}_{{{28}}} + 0.{\text{234X}}_{{{29}}} \\ &+ 0.{\text{276X}}_{{{3}0}} + 0.{19}0{\text{X}}_{{{31}}} \\ \end{aligned}$$

$$\begin{aligned} {\text{Y}}_{{2}} = \,& 0.0{\text{23X}}_{{1}} - 0.{1}0{\text{9X}}_{{2}} + 0.{\text{122X}}_{{3}} + 0.{\text{315X}}_{{4}} + 0.{\text{157X}}_{{5}} + 0.{\text{311X}}_{{6}} + 0.{\text{117X}}_{{7}} + 0.{\text{182X}}_{{8}} + 0.{2}00{\text{X}}_{{9}} + 0.{\text{293X}}_{{{1}0}} \\ & + 0.{\text{198X}}_{{{11}}} + 0.{2}0{\text{5X}}_{{{12}}} - 0.{1}00{\text{X}}_{{{13}}} - 0.{14}0{\text{X}}_{{{14}}} - 0.{1}0{\text{6X}}_{{{15}}} + 0.{\text{289X}}_{{{16}}} + 0.{2}00{\text{X}}_{{{17}}} - 0.{\text{123X}}_{{{18}}} - 0.{\text{151X}}_{{{19}}} \\ &- 0.{\text{158X}}_{{{2}0}} - 0.{\text{171X}}_{{{21}}} + 0.{\text{245X}}_{{{22}}} - 0.0{\text{11X}}_{{{23}}} - 0.{\text{221X}}_{{{24}}} - 0.0{\text{85X}}_{{{25}}} + 0.{\text{153X}}_{{{26}}} + 0.0{\text{53X}}_{{{27}}} - 0.{\text{217X}}_{{{28}}} \\ &- 0.{\text{167X}}_{{{29}}} - 0.{\text{139X}}_{{{3}0}} - 0.0{\text{69X}}_{{{31}}} \\ \end{aligned}$$

$$\begin{aligned} {\text{Y}}_{{3}} =\, & 0.0{\text{46X}}_{{1}} - 0.0{\text{14X}}_{{2}} + 0.{\text{195X}}_{{3}} - 0.0{\text{65X}}_{{4}} - 0.{\text{161X}}_{{5}} + 0.{1}0{\text{1X}}_{{6}} - 0.{\text{277X}}_{{7}} - 0.0{9}0{\text{X}}_{{8}} - 0.{\text{145X}}_{{9}} \\& - 0.{\text{212X}}_{{{1}0}} - 0.{\text{241X}}_{{{11}}} - 0.{\text{275X}}_{{{12}}} + 0.{1}0{\text{2X}}_{{{13}}} + 0.{\text{111X}}_{{{14}}} - 0.{\text{158X}}_{{{15}}} + 0.{\text{274X}}_{{{16}}} + 0.{\text{311X}}_{{{17}}} - 0.0{\text{21X}}_{{{18}}} - 0.00{\text{7X}}_{{{19}}} + 0.{\text{225X}}_{{{2}0}} \\& + 0.0{\text{78X}}_{{{21}}} + 0.{\text{275X}}_{{{22}}} + 0.{15}0{\text{X}}_{{{23}}} - 0.{17}0{\text{X}}_{{{24}}} - 0.{\text{147X}}_{{{25}}} + 0.{\text{335X}}_{{{26}}} + 0.{\text{167X}}_{{{27}}} + 0.0{\text{51X}}_{{{28}}} - 0.0{\text{49X}}_{{{29}}} + 0.0{\text{88X}}_{{{3}0}} + 0.{\text{236X}}_{{{31}}} \\ \end{aligned}$$

$$\begin{aligned} {\text{Y}}_{{4}} = \,& - 0.{\text{126X}}_{{1}} - 0.{\text{166X}}_{{2}} + 0.{\text{242X}}_{{3}} - 0.0{9}0{\text{X}}_{{4}} - 0.{\text{152X}}_{{5}} + 0.{\text{157X}}_{{6}} + 0.{\text{139X}}_{{7}} \\& + 0.{\text{375X}}_{{8}} - 0.{\text{155X}}_{{9}} + 0.0{\text{37X}}_{{{1}0}} - 0.0{\text{55X}}_{{{11}}} - 0.{17}0{\text{X}}_{{{12}}} + 0.{\text{367X}}_{{{13}}} - 0.00{\text{7X}}_{{{14}}} - 0.0{\text{99X}}_{{{15}}} + 0.0{\text{12X}}_{{{16}}} + 0.{\text{117X}}_{{{17}}} + 0.0{\text{83X}}_{{{18}}} + 0.{\text{226X}}_{{{19}}} \\& - 0.{16}0{\text{X}}_{{{2}0}} + 0.0{\text{14X}}_{{{21}}} + 0.0{7}0{\text{X}}_{{{22}}} - 0.{\text{192X}}_{{{23}}} + 0.0{\text{39X}}_{{{24}}} + 0.{55}0{\text{X}}_{{{25}}} + 0.000{\text{X}}_{{{26}}} - 0.0{\text{24X}}_{{{27}}} + 0.00{\text{7X}}_{{{28}}} \\& + 0.0{\text{69X}}_{{{29}}} + 0.0{\text{12X}}_{{{3}0}} - 0.{18}0{\text{X}}_{{{31}}} \\ \end{aligned}$$

$$\begin{aligned} {\text{Y}}_{{5}} = \,& 0.{\text{172X}}_{{1}} - 0.{\text{188X}}_{{2}} + 0.0{\text{38X}}_{{3}} - 0.{1}00{\text{X}}_{{4}} + 0.{2}0{\text{8X}}_{{5}} + 0.0{\text{17X}}_{{6}} - 0.0{\text{17X}}_{{7}} - 0.{2}0{\text{4X}}_{{8}} + 0.0{\text{68X}}_{{9}} \\& + 0.0{1}0{\text{X}}_{{{1}0}} - 0.0{2}0{\text{X}}_{{{11}}} + 0.{\text{137X}}_{{{12}}} + 0.{\text{433X}}_{{{13}}} + 0.{21}0{\text{X}}_{{{14}}} + 0.{\text{313X}}_{{{15}}} + 0.0{\text{44X}}_{{{16}}} + 0.{11}0{\text{X}}_{{{17}}} + 0.{\text{174X}}_{{{18}}} \\& - 0.{\text{114X}}_{{{19}}} - 0.{\text{168X}}_{{{2}0}} - 0.{\text{253X}}_{{{21}}} - 0.0{\text{97X}}_{{{22}}} - 0.{\text{221X}}_{{{23}}} + 0.{\text{259X}}_{{{24}}} - 0.{21}0{\text{X}}_{{{25}}} + 0.{\text{318X}}_{{{26}}} \\ &+ 0.{\text{138X}}_{{{27}}} - 0.00{\text{6X}}_{{{28}}} + 0.{\text{168X}}_{{{29}}} + 0.0{\text{55X}}_{{{3}0}} - 0.{12}0{\text{X}}_{{{31}}} \\ \end{aligned}$$

$$\begin{aligned} {\text{Y}}_{{6}} = \,& 0.{\text{181X}}_{{1}} + 0.{19}0{\text{X}}_{{2}} + 0.0{\text{98X}}_{{3}} + 0.0{\text{87X}}_{{4}} - 0.{\text{138X}}_{{5}} - 0.0{\text{16X}}_{{6}} - 0.00{\text{3X}}_{{7}} - 0.{\text{135X}}_{{8}} \\& - 0.0{\text{66X}}_{{9}} - 0.0{\text{29X}}_{{{1}0}} - 0.0{7}0{\text{X}}_{{{11}}} + 0.00{\text{3X}}_{{{12}}} - 0.0{\text{62X}}_{{{13}}} - 0.{\text{229X}}_{{{14}}} + 0.0{\text{43X}}_{{{15}}} + 0.{\text{165X}}_{{{16}}} - 0.{\text{115X}}_{{{17}}} - 0.{\text{165X}}_{{{18}}} + 0.0{1}0{\text{X}}_{{{19}}} \\& - 0.{\text{213X}}_{{{2}0}} - 0.0{8}0{\text{X}}_{{{21}}} + 0.{\text{243X}}_{{{22}}} + 0.{\text{271X}}_{{{23}}} + 0.{3}0{\text{9X}}_{{{24}}} + 0.0{\text{73X}}_{{{25}}} - 0.{2}0{\text{4X}}_{{{26}}} + 0.{\text{516X}}_{{{27}}} + 0.0{\text{96X}}_{{{28}}} \\& + 0.{\text{133X}}_{{{29}}} + 0.{\text{121X}}_{{{3}0}} - 0.{\text{322X}}_{{{31}}} \\ \end{aligned}$$

$$\begin{aligned} {\text{Y}}_{{7}} = \,& - 0.{\text{146X}}_{{1}} - 0.0{\text{62X}}_{{2}} - 0.0{\text{83X}}_{{3}} - 0.0{\text{53X}}_{{4}} + 0.{\text{137X}}_{{5}} + 0.00{\text{4X}}_{{6}} - 0.{\text{163X}}_{{7}} \\& + 0.{2}0{\text{7X}}_{{8}} + 0.{\text{134X}}_{{9}} + 0.{\text{166X}}_{{{1}0}} - 0.0{\text{82X}}_{{{11}}} - 0.00{\text{6X}}_{{{12}}} + 0.{\text{139X}}_{{{13}}} - 0.{\text{286X}}_{{{14}}} + 0.{\text{644X}}_{{{15}}} \\& + 0.{\text{139X}}_{{{16}}} - 0.0{\text{95X}}_{{{17}}} - 0.{2}0{\text{1X}}_{{{18}}} + 0.{\text{316X}}_{{{19}}} + 0.{2}0{\text{8X}}_{{{2}0}} + 0.{\text{213X}}_{{{21}}} - 0.00{\text{2X}}_{{{22}}} + 0.{\text{149X}}_{{{23}}} \\& - 0.0{\text{49X}}_{{{24}}} + 0.0{\text{17X}}_{{{25}}} + 0.0{\text{96X}}_{{{26}}} + 0.0{\text{84X}}_{{{27}}} - 0.0{\text{21X}}_{{{28}}} - 0.0{\text{52X}}_{{{29}}} - 0.0{3}0{\text{X}}_{{{3}0}} + 0.0{\text{81X}}_{{{31}}} \\ \end{aligned}$$

$$\begin{aligned} {\text{Y}}_{{8}} =\, & 0.{\text{187X}}_{{1}} + 0.{\text{358X}}_{{2}} + 0.0{\text{62X}}_{{3}} + 0.0{\text{43X}}_{{4}} - 0.0{\text{72X}}_{{5}} - 0.{\text{157X}}_{{6}} + 0.{\text{155X}}_{{7}} - 0.0{\text{61X}}_{{8}} - 0.0{2}0{\text{X}}_{{9}} \\& - 0.0{\text{43X}}_{{{1}0}} - 0.0{\text{36X}}_{{{11}}} - 0.0{4}0{\text{X}}_{{{12}}} + 0.{\text{182X}}_{{{13}}} + 0.0{\text{41X}}_{{{14}}} + 0.{\text{216X}}_{{{15}}} - 0.0{\text{34X}}_{{{16}}} + 0.{\text{296X}}_{{{17}}} - 0.{\text{174X}}_{{{18}}} - 0.{\text{186X}}_{{{19}}} \\& + 0.0{\text{15X}}_{{{2}0}} + 0.0{\text{12X}}_{{{21}}} + 0.0{\text{65X}}_{{{22}}} + 0.{\text{321X}}_{{{23}}} + 0.{\text{158X}}_{{{24}}} + 0.{\text{138X}}_{{{25}}} + 0.{1}0{\text{1X}}_{{{26}}} - 0.{5}0{\text{2X}}_{{{27}}} - 0.{\text{143X}}_{{{28}}} - 0.{\text{222X}}_{{{29}}} \\& - 0.{1}0{\text{6X}}_{{{3}0}} - 0.{2}0{\text{1X}}_{{{31}}} \\ \end{aligned}$$

Normalizing the original variables and using SPSS to compute variables and calculate the principal componentsY1, Y2, Y3, Y4, Y5, Y6, Y7 and Y8. Taking the variance contribution rate corresponding to each principal component as the weight, the principal component scores and the corresponding weights were linearly weighted to construct a comprehensive evaluation function of different habitats and batches of Leonuri Herba:

$${\text{Y}} = 0.{3}0{\text{1 Y}}_{{1}} + 0.{\text{192Y}}_{{2}} + 0.{\text{131Y}}_{{3}} + 0.0{\text{78Y}}_{{4}} + 0.0{\text{52Y}}_{{5}} + 0.0{\text{43Y}}_{{6}} + 0.0{\text{38Y}}_{{7}} + 0.0{\text{34Y}}_{{8}}$$

The comprehensive evaluation scores of Leonuri Herba from different origins and batches were calculated from the above formula are shown in Table 4. The higher of the score, the higher content of active ingredient in the sample. The comprehensive score was greater than 0. The results showed that batch S8 (from Hubei at seedling stage) had the highest comprehensive score, followed by batch S4 (from Guangdong at seedling stage), S15 (Henan) S10 (from Hubei at seedling stage) and S13 (Henan). The above results showed that the quality of three batches at seedling stage was better than the others. The chemical components of Leonuri Herba produced in Hubei and Henan are relatively higher than other origins.

**Table 4:** Principal component scores, comprehensive evaluation, and fine sorting of Leonuri Herba

Batch no	Code	Region	Main components								Comprehensive score	Order
			Y1	Y2	Y3	Y4	Y5	Y6	Y7	Y8		
S8	HB5	Hubei	0.25	1.42	2.31	− 0.79	− 0.57	0.17	− 0.63	1.32	1.89	1
S4	GD1	Guangdong	0.10	3.00	1.18	− 0.83	− 0.22	0.36	0.30	0.96	1.71	2
S15	HN5	Henan	7.64	− 2.62	2.86	− 1.67	− 1.85	− 1.33	1.18	− 0.44	1.53	3
S10	HB7	Hubei	− 0.17	1.28	2.75	0.92	2.17	− 1.80	− 0.35	0.73	1.42	4
S13	HN3	Henan	0.19	0.02	− 2.69	− 1.62	− 1.08	0.46	0.44	1.73	1.33	5
S14	HN4	Henan	6.42	− 2.66	− 1.50	1.77	1.43	1.37	− 0.32	− 0.40	1.12	6
S23	YN4	Yunnan	0.12	− 0.32	-0.12	0.30	− 0.96	− 0.15	− 0.13	0.98	0.93	7
S7	GD3	Guangdong	− 0.62	1.96	1.03	− 0.30	− 0.32	− 0.31	− 0.73	0.45	0.73	8
S5	HB4	Hubei	− 0.89	0.50	0.37	5.90	− 2.15	− 0.29	1.05	0.33	0.61	9
S19	AH4	Anhui	− 0.81	0.22	− 0.55	− 0.15	− 0.75	1.74	− 0.57	0.52	0.29	10
S9	HB6	Hubei	0.88	4.57	2.03	− 0.35	− 0.59	2.34	0.76	− 1.36	0.16	11
S27	SC2	Sichuang	− 0.86	1.65	− 2.85	− 0.49	2.15	− 1.50	1.88	0.22	0.02	12

### Result of quantitative analysis

**Fig. 5:**
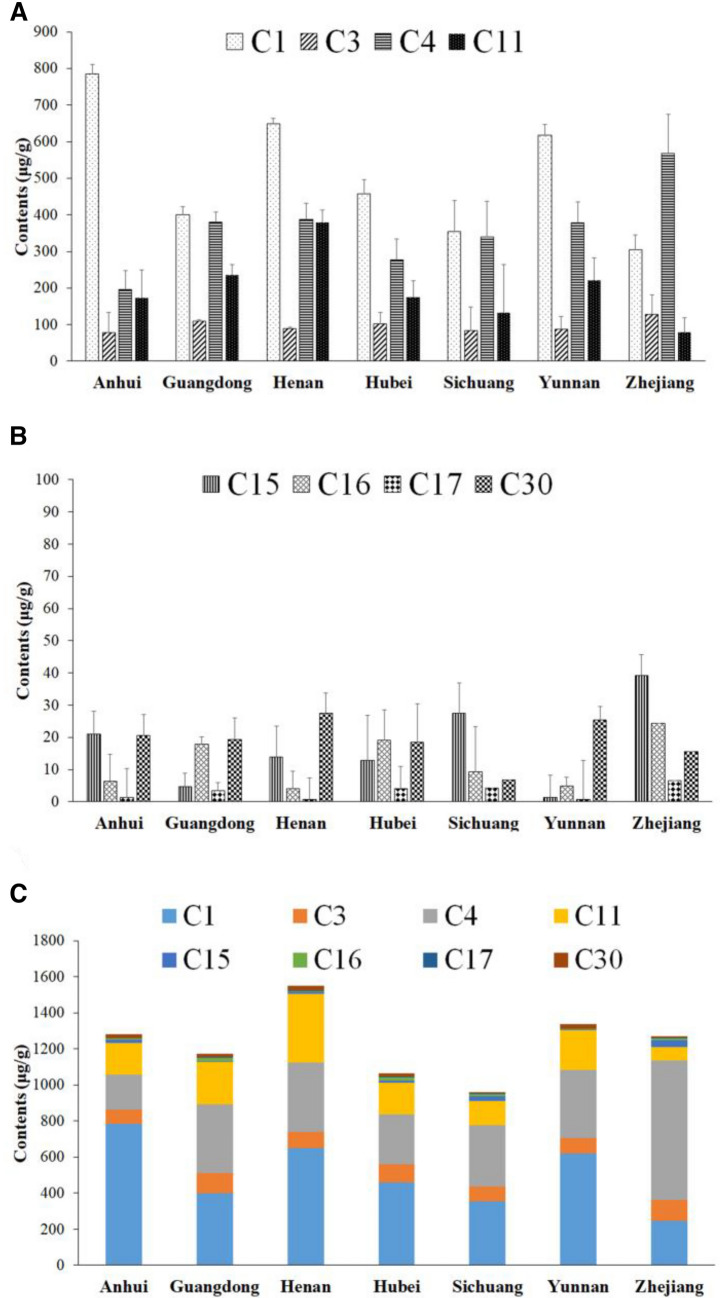
The contents of 8 components in the 30 samples from different regions. **A** The content of Leonurine (C1), 4',5-dihydroxy-7-methoxyflavone (C3), rutin (C4) and hyperoside (C11). **B** The content of apigenin(C15), quercetin(C16), kaempferol (C17) and salicylic acid (C30). **C** The content of combination of 8 components in 30 samples

The LOD was calculated according to the signal-to-noise ratio of 3:1; the LOQ was calculated according to the signal-to-noise ratio of 10:1, and the results were shown in (Additional file 2: Table S1). The precision test results showed that the precision of the instrument was good; the stability test results showed that the test solution had a good stability within 24 h after preparation; the repeatability test results showed that the method had a good repeatability; the results of the sample recovery rate showed that the accuracy was good (Additional file 3: Table S2). The results of 30 batches of Leonuri Herba from different origins are shown in Table 5. The detailed content trends of eight analytes in the 30 samples from different origins are exhibited in Fig. 5. In Fig. 5A, the results showed that Leonuri Herba from Henan had the highest content in hyperoside (C11) and salicylic acid (C30). The content of leonurine (C1) in Anhui was the highest while the content of rutin (C4) in Zhejiang was the highest. For apigenin (C15), quercetin(C16) and kaempferol (C17), Hubei, Henan and Sichuan had similar result (Fig. 5B). By adding all the eight analytes together, the total content of Henan is the highest (Fig. 5C).

**Table 5:** The Contents (μg/g) of the eight marker compounds in 30 samples of Leonuri Herba

Batch no	Region	C1	C3	C4	C11	C15	C16	C17	C30
S1	Hubei	100.41 ± 12.14	68.06 ± 5.73	213.93 ± 13.21	42.41 ± 4.20	53.39 ± 3.17	3.25 ± 1.22	–	1.34 ± 0.57
S2	Hubei	583.07 ± 13.24	98.82 ± 0.48	316.51 ± 11.52	211.54 ± 15.53	6.65 ± 0.67	18.33 ± 4.05	4.82 ± 0.71	7.47 ± 1.61
S3	Hubei	434.12 ± 11.17	52.34 ± 6.79	93.15 ± 19.07	103.12 ± 18.30	2.71 ± 0.47	4.47 ± 1.92	0.094 ± 0.024	10.68 ± 1.70
S4	Guangdong	410.38 ± 9.73	113.29 ± 4.73	422.06 ± 15.45	279.04 ± 12.10	3.68 ± 0.15	22.57 ± 3.17	4.16 ± 0.36	32.90 ± 0.80
S5	Hubei	524.77 ± 13.19	118.37 ± 8.96	199.12 ± 12.22	153.81 ± 9.93	7.11 ± 0.12	10.41 ± 2.34	2.20 ± 0.39	5.58 ± 0.36
S6	Guangdong	484.28 ± 11.23	111.96 ± 2.07	265.15 ± 32.64	159.25 ± 22.00	3.55 ± 0.26	15.90 ± 3.29	3.59 ± 0.57	6.74 ± 1.09
S7	Guangdong	308.20 ± 17.56	105.76 ± 8.42	453.84 ± 14.86	267.73 ± 17.56	7.02 ± 0.95	15.42 ± 1.78	2.41 ± 0.23	18.14 ± 0.53
S8	Hubei	633.78 ± 15.77	134.32 ± 10.86	223.65 ± 14.78	220.24 ± 15.85	4.10 ± 0.20	25.23 ± 3.32	5.74 ± 0.16	58.33 ± 1.36
S9	Hubei	445.87 ± 12.38	138.33 ± 4.80	595.55 ± 29.85	281.67 ± 9.66	6.58 ± 0.11	56.06 ± 4.91	8.03 ± 0.31	41.50 ± 2.01
S10	Hubei	481.32 ± 4.22	108.57 ± 2.93	295.44 ± 8.95	204.89 ± 9.31	8.85 ± 0.29	16.27 ± 2.56	3.44 ± 0.43	4.24 ± 0.50
S11	Henan	712.63 ± 9.89	82.27 ± 3.93	543.24 ± 13.73	476.30 ± 15.05	–	4.19 ± 0.14	0.95 ± 0.17	14.79 ± 0.74
S12	Henan	774.25 ± 16.37	90.91 ± 8.31	563.89 ± 11.56	504.91 ± 31.63	–	2.64 ± 0.73	1.06 ± 0.71	17.40 ± 2.65
S13	Henan	584.71 ± 11.23	92.79 ± 10.20	412.14 ± 10.42	429.45 ± 6.88	28.09 ± 1.59	5.38 ± 1.46	0.021 ± 0.0019	57.28 ± 3.87
S14	Henan	590.93 ± 36.27	87.64 ± 3.55	216.92 ± 16.25	277.67 ± 18.09	11.98 ± 0.84	1.46 ± 0.13	–	22.80 ± 1.52
S15	Henan	584.71 ± 6.50	89.43 ± 4.07	197.06 ± 11.02	208.28 ± 15.38	1.36 ± 0.33	7.04 ± 1.86	1.18 ± 0.0059	25.01 ± 1.12
S16	Anhui	903.50 ± 17.53	47.57 ± 6.09	78.95 ± 11.99	62.36 ± 9.92	14.23 ± 1.21	–	–	12.73 ± 2.62
S17	Anhui	626.83 ± 13.69	90.99 ± 2.82	266.77 ± 12.35	253.48 ± 12.00	36.89 ± 1.61	2.58 ± 0.18	0.46 ± 0.051	23.55 ± 1.67
S18	Anhui	1021.49 ± 14.23	38.42 ± 2.83	146.53 ± 11.99	63.34 ± 5.38	29.56 ± 0.64	–	–	7.73 ± 0.71
S19	Anhui	584.86 ± 11.31	133.85 ± 2.95	292.31 ± 16.28	315.71 ± 12.43	3.73 ± 0.60	10.18 ± 1.57	2.08 ± 0.31	38.27 ± 2.71
S20	Yunnan	519.11 ± 16.82	84.36 ± 0.64	282.24 ± 19.08	312.05 ± 17.05	1.25 ± 0.27	4.69 ± 0.32	0.36 ± 0.035	19.76 ± 1.63
S21	Yunnan	834.18 ± 12.17	64.28 ± 1.48	699.53 ± 23.25	16.80 ± 2.46	2.77 ± 0.32	–	–	30.90 ± 3.47
S22	Yunnan	436.10 ± 18.56	67.37 ± 8.79	240.06 ± 25.27	271.45 ± 18.89	0.76 ± 0.049	3.53 ± 0.44	0.11 ± 0.021	14.03 ± 1.63
S23	Yunnan	684.29 ± 5.69	131.20 ± 8.22	291.34 ± 17.45	284.58 ± 21.66	0.75 ± 0.040	6.19 ± 0.13	1.79 ± 0.41	37.25 ± 3.25
S24	Zhejiang	317.46 ± 10.69	50.38 ± 5.70	275.95 ± 19.22	110.20 ± 9.36	45.01 ± 1.89	–	–	5.49 ± 0.37
S25	Zhejiang	171.68 ± 1.67	183.50 ± 4.56	1268.47 ± 11.15	46.09 ± 3.61	22.72 ± 0.42	12.15 ± 3.67	2.98 ± 0.34	15.81 ± 0.20
S26	Sichuang	163.08 ± 10.02	101.56 ± 3.67	408.43 ± 8.39	16.25 ± 0.82	23.29 ± 0.34	–	–	–
S27	Sichuang	177.20 ± 9.54	150.39 ± 5.78	782.02 ± 11.48	394.56 ± 16.30	64.31 ± 2.45	18.49 ± 2.03	4.18 ± 0.20	–
S28	Sichuang	802.61 ± 11.85	26.53 ± 1.39	102.46 ± 21.16	57.76 ± 10.27	16.92 ± 1.83	–	–	–
S29	Sichuang	274.72 ± 19.67	56.79 ± 8.34	64.12 ± 1.62	64.22 ± 2.08	5.80 ± 0.10	0.08 ± 0.05	–	6.89 ± 1.44
S30	Zhejiang	422.63 ± 6.19	147.58 ± 3.13	159.56 ± 9.73	78.62 ± 6.17	50.18 ± 9.94	36.54 ± 5.12	10.12 ± 0.86	25.37 ± 1.47

Values represent means ± SD, n = 3; “–”, below the detection limit

C1: leonurine, C3: 4',5-dihydroxy-7-methoxyflavone, C4: rutin, C11: hyperoside, C15: apigenin, C16: quercetin, C17: kaempferol, C30: salicylic acid, same below

In summary, the quality of Leonuri Herba at seedling stage is better than the mature ones. Apart from the quality of medicinal part, Leonuri Herba from different origins are mainly clustered into four big categories: Henan and Yunnan are grouped together; Zhejiang and Sichuan are grouped together; Anhui is grouped as one group; Guangdong and Hubei (at seedlings and flowering stage) are grouped together while Hubei (at mature stage) is in different group due to growing stage difference. The comprehensive evaluation analysis showed that the quality of Leonuri Herba at seedling stage was good. And the quality of Leonuri Herba in Henan was relatively good when compared to other origins. However, the quality difference between stems and leaves from different origins and different growing stage is not very clear. Further studies should be conducted to address this issue. Laser microdissection combined with chromatographic analysis could be one of the powerful tools to investigate the chemical composition distribution and change in different growing stages of Leonuri Herba. This may also help to unify the standard of Leonuri Herba so as to decrease the confusion in markets.

## Conclusion

The current study revealed a clear correlation between quality and geographical origin of Leonuri Herba. According to the correlation, the raw materials of Leonuri Herba from different regions can be formulated in a certain proportion and used for its traditional Chinese medicine preparations, thereby ensuring the safety, stability and effectiveness of clinical application; however, extensive pharmacological research is still required to address this issue. In a word, the proposed fingerprint will be important for authentication; the developed quantitative method will be useful for quality control of Leonuri Herba and its preparations; the revealed correlation could be significant for conservation and utilization of Leonuri Herba as a natural resource.

## Supplementary Information

**Additional file 1.** Photograph of Leonuri Herba at seedling stage (upper) and mature stage (lower).

**Additional file 2: Table S1.** Calibration curves, correlation factors, linear ranges, LOD and LOQ for the eight compounds.

**Additional file 3: Table S2.** The RSD values of precisions, reproducibility, stability and recovery for the eight compounds.

## Data Availability

The data used to support the current study are available from the corresponding author on reasonable request.

## Abbreviations

BPC: Base peak chromatogram
LOD: Limit of detection
LOQ: Limit of quantification
MF: Molecular formula
MS: Mass spectrometry
PCA: Principal component analysis
TCM: Traditional Chinese medicine
UHPLC: Ultra-high performance liquid chromatography
OPLS-DA: Orthogonal partial least squares discriminant analysis
