# Supplementary figures and images for: Correlation between quality and geographical origins of Leonuri Herba revealed by the qualitative fingerprint profiling and quantitative determination of chemical components
Source: Chin Med. 2022 Apr 12;17:46. doi: 10.1186/s13020-022-00592-w (PMC9003958; doi:10.1186/s13020-022-00592-w)

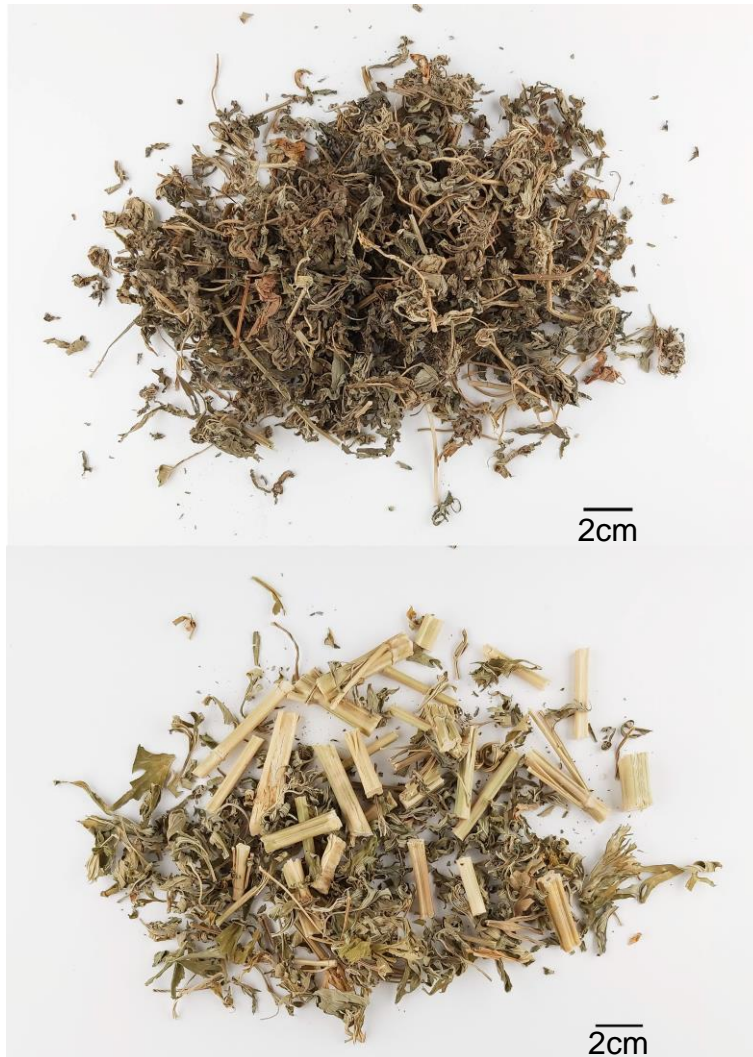

Addition  
File1  
Lam et

Supplement: Supplementary file 1 — Additional file 1. Photograph of Leonuri Herba at seedling stage (upper) and mature stage (lower). [file 13020_2022_592_MOESM1_ESM.pdf]
