# Supplementary material for: Correlation between quality and geographical origins of Leonuri Herba revealed by the qualitative fingerprint profiling and quantitative determination of chemical components
Source: Chin Med. 2022 Apr 12;17:46. doi: 10.1186/s13020-022-00592-w (PMC9003958; doi:10.1186/s13020-022-00592-w)
Supplement: Supplementary file 2 — Additional file 2: Table S1. Calibration curves, correlation factors, linear ranges, LOD and LOQ for the eight compounds. [file 13020_2022_592_MOESM2_ESM.docx]

Additional file 2

Table S1 Calibration curves, correlation factors, linear ranges, LOD and LOQ for the eight compounds

| Compounds | Linear regression data | | | LOD | LOQ |
| --- | --- | --- | --- | --- | --- |
|  | Regression equation | R^2^ | Linear Range  (µg/mL) | (µg/mL) | (µg/mL) |
| C1 | Y = 85728.14 X + 937869.50 | 0.9938 | 12.60 ~ 67.79 | 1.47 | 4.88 |
| C3 | Y = 676127.60 X + 3043706.13 | 0.9929 | 3.00 ~ 16.14 | 0.34 | 1.14 |
| C4 | Y = 58192.40 X + 1067111.57 | 0.9908 | 15.00 ~ 80.70 | 1.07 | 3.56 |
| C11 | Y = 67933.71 X + 726171.25 | 0.9930 | 7.20 ~ 38.74 | 0.75 | 2.51 |
| C15 | Y = 455783.46 X + 666138.39 | 0.9948 | 1.50 ~ 8.07 | 0.42 | 1.40 |
| C16 | Y = 165593.12 X + 432482.55 | 0.9964 | 3.60 ~ 19.37 | 0.62 | 2.05 |
| C17 | Y = 412855.11 X + 393133.52 | 0.9963 | 1.50 ~ 8.07 | 0.42 | 1.39 |
| C30 | Y = 325935.92 X + 547324.16 | 0.9967 | 2.40 ~ 12.91 | 0.65 | 2.16 |

Remarks：C1: leonurine, C3: 4',5-dihydroxy-7-methoxyflavone, C4: rutin, C11: hyperoside, C15: apigenin, C16: quercetin, C17: kaempferol,

C30: salicylic acid, same below.
