# Supplementary material for: Correlation between quality and geographical origins of Leonuri Herba revealed by the qualitative fingerprint profiling and quantitative determination of chemical components
Source: Chin Med. 2022 Apr 12;17:46. doi: 10.1186/s13020-022-00592-w (PMC9003958; doi:10.1186/s13020-022-00592-w)
Supplement: Supplementary file 3 — Additional file 3: Table S2. The RSD values of precisions, reproducibility, stability and recovery for the eight compounds. [file 13020_2022_592_MOESM3_ESM.docx]

Additional file 3

Table S2 The RSD values of precisions, reproducibility, stability and recovery for the eight compounds

| Compounds | intra- and inter-day Precisions | | Reproducibility | Stability | Recovery (n=3) | |
| --- | --- | --- | --- | --- | --- | --- |
|  | Intra-day（n=3）RSD (%) | Inter-day（n=3）RSD (%) | (n= 6)  RSD (%) | (24 h, n= 6) RSD (%) | Mean | RSD (%) |
| C1 | 0.77 | 1.56 | 1.96 | 3.71 | 97.88 | 4.44 |
| C3 | 0.77 | 2.84 | 1.88 | 2.80 | 100.09 | 2.14 |
| C4 | 2.97 | 7.21 | 1.96 | 5.71 | 97.73 | 4.41 |
| C11 | 3.48 | 5.78 | 4.14 | 3.19 | 101.82 | 4.69 |
| C15 | 1.67 | 0.28 | 4.65 | 7.62 | 97.15 | 0.63 |
| C16 | 3.92 | 5.67 | 3.65 | 5.28 | 102.55 | 1.81 |
| C17 | 0.98 | 3.14 | 4.33 | 6.76 | 96.52 | 1.98 |
| C30 | 0.50 | 4.56 | 3.20 | 1.49 | 98.56 | 2.66 |

Remarks：C1: leonurine, C3: 4',5-dihydroxy-7-methoxyflavone, C4: rutin, C11: hyperoside, C15: apigenin, C16: quercetin, C17: kaempferol,

C30: salicylic acid, same below.
